# CandiSSR: An Efficient Pipeline used for Identifying Candidate Polymorphic SSRs Based on Multiple Assembled Sequences

**DOI:** 10.3389/fpls.2015.01171

**Published:** 2016-01-07

**Authors:** En-Hua Xia, Qiu-Yang Yao, Hai-Bin Zhang, Jian-Jun Jiang, Li-Ping Zhang, Li-Zhi Gao

**Affiliations:** ^1^Plant Germplasm and Genomics Center, Germplasm Bank of Wild Species in Southwest China, Kunming Institute of Botany, Chinese Academy of SciencesKunming, China; ^2^University of Chinese Academy of SciencesBeijing, China

**Keywords:** microsatellites, transferability, polymorphic SSR, CandiSSR, multiple assembled genomes, multiple assembled transcriptomes

## Abstract

Simple sequence repeats (SSRs), also known as microsatellites, are ubiquitous short tandem duplications commonly found in genomes and/or transcriptomes of diverse organisms. They represent one of the most powerful molecular markers for genetic analysis and breeding programs because of their high mutation rate and neutral evolution. However, traditionally experimental screening of the SSR polymorphic status and their subsequent applicability to genetic studies are extremely labor-intensive and time-consuming. Thankfully, the recently decreased costs of next generation sequencing and increasing availability of large genome and/or transcriptome sequences have provided an excellent opportunity and sources for large-scale mining this type of molecular markers. However, current tools are limited. Thus we here developed a new pipeline, CandiSSR, to identify candidate polymorphic SSRs (PolySSRs) based on the multiple assembled sequences. The pipeline allows users to identify putative PolySSRs not only from the transcriptome datasets but also from multiple assembled genome sequences. In addition, two confidence metrics including standard deviation and missing rate of the SSR repetitions are provided to systematically assess the feasibility of the detected PolySSRs for subsequent application to genetic characterization. Meanwhile, primer pairs for each identified PolySSR are also automatically designed and further evaluated by the global sequence similarities of the primer-binding region, ensuring the successful rate of the marker development. Screening rice genomes with CandiSSR and subsequent experimental validation showed an accuracy rate of over 90%. Besides, the application of CandiSSR has successfully identified a large number of PolySSRs in the *Arabidopsis* genomes and *Camellia* transcriptomes. CandiSSR and the PolySSR marker sources are publicly available at: http://www.plantkingdomgdb.com/CandiSSR/index.html.

## Introduction

Simple sequence repeats (SSRs; also called microsatellites), containing repetitive sequences of 1–6 bp in length, have been extensively found in both the coding and non-coding sequences of eukaryotic and prokaryotic genomes (Tautz and Renz, 1984; Gupta et al., 1996; Li et al., 2002; Zhang et al., 2004). They are broadly applied in various areas of genetic studies including the evaluation of genetic variation (Kashi et al., 1997), construction of genetic linkage maps (Jones et al., 2002), QTL analysis (Mei et al., 2004; Minamiyama et al., 2007), positional cloning and molecular marker-assisted selection in plant and animal breeding programs (Mohan et al., 1997; Collard and Mackill, 2008). In recent years, genomic microsatellites (gSSR) have attracted more attention owing to high level of polymorphisms, reproducibility and abundance in plant genomes (Jones et al., 1997; Uzunova and Ecke, 1999). Compared to gSSRs, expressed sequence tag (EST)-SSRs belong to the transcribed DNA regions and exhibit potential advantages due to their high across-species transferability rate and more generally consistent amplification efficiency (Scott et al., 2000; Gupta et al., 2003). Moreover, the majority of EST-SSR loci are present in functional genes, indicating these markers could possibly be associated with some significant phenotypes.

Owing to the recent rapid development of next-generation sequencing techniques, hundreds of genomes and transcriptomes of commercially or experimentally important organisms have been sequenced (Arabidopsis Genome Initiative, 2000; Goff et al., 2002; Grabherr et al., 2011). Accordingly, thousands of gSSRs and EST-SSRs of these species were also collected (Aranzana et al., 2003; Xia et al., 2014). However, due to a low efficiency of the traditional laboratory assessment for the SSR polymorphic status and their subsequent applicability to genetic studies, fewer available polymorphic SSRs (PolySSRs) are currently identified, which largely hampers the fairly urgent needs for efficient employment of the abundant SSR sources toward genetic studies and breeding efforts.

Simple sequence repeats marker development mainly consists of three separate steps: SSR discovery, primer design and polymorphic survey in representative population or individuals. Traditional approaches for SSR development were costly and consists of time-consuming procedures such as SSR-enriched libraries construction and candidate clone sequencing (Sargent et al., 2003) as well as polyacrylamide gel electrophoresis and/or fluorescent capillary electrophoresis (Bassil et al., 2005). More recently, alternative methods of SSR development based on mining the already available genomic and/or transcriptomic sequence data (Wen et al., 2010; Lee et al., 2014) turned out to be more economical and efficient. Several computational tools have also been developed such as MISA (Thiel et al., 2003), SSR Primer (Robinson et al., 2004), and SSR Locator (da Maia et al., 2008). However, SSRs discovered by these tools are still required to manually screen their polymorphic status because of these tools have not yet integrated a computational solution for systematically assessment the SSR polymorphic status. Thus an easy-to-use software that integrates SSR discovery, primer design as well as *in silico* assessment of the SSRs polymorphic status based on existing sequence data from multiple individuals or species will surely greatly meet the urgent demands.

**FIGURE 1:**
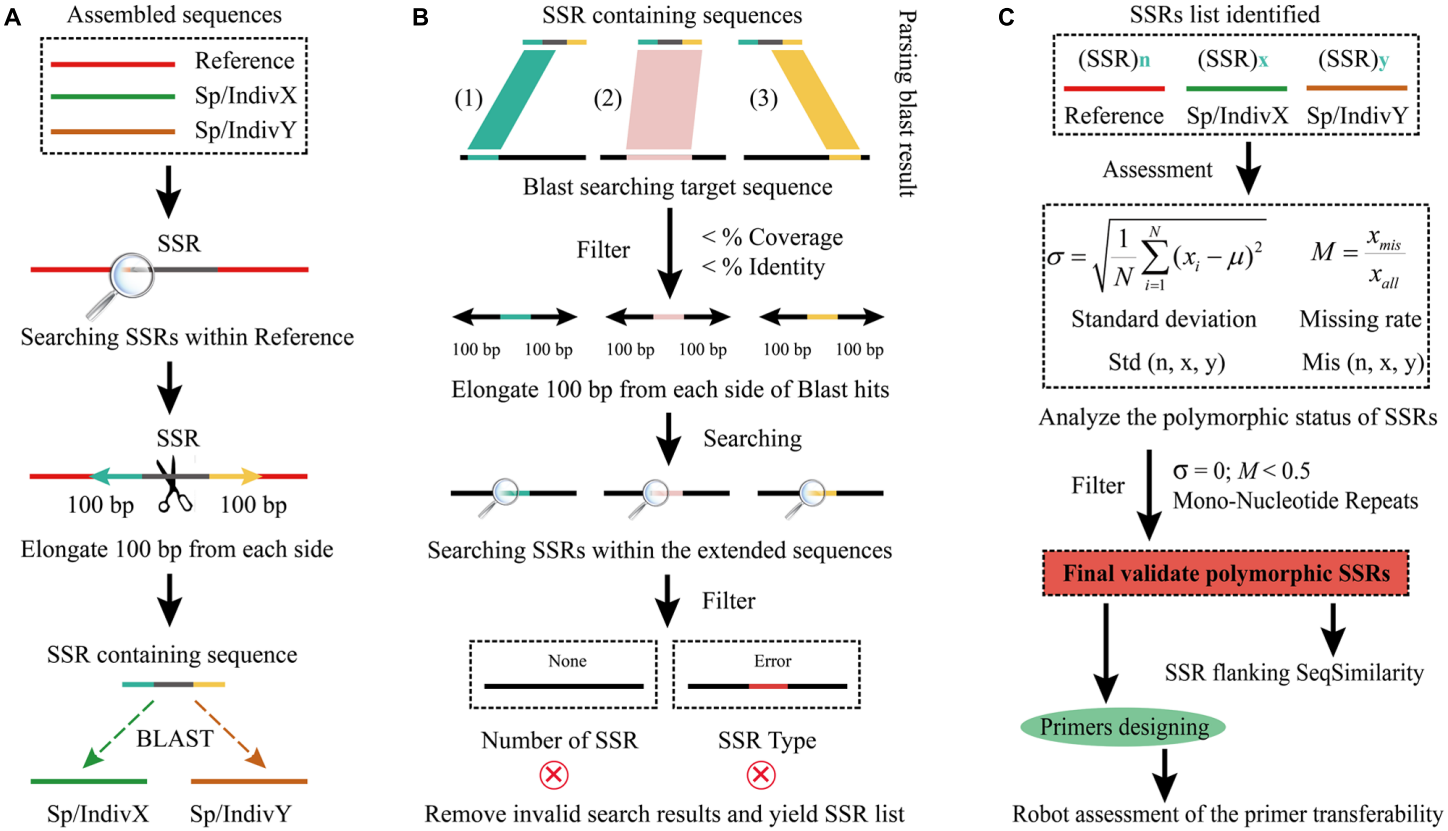
**Flowchart of the CandiSSR pipeline.** **(A)** Data preparation and Blast searches; **(B)** Blast result parsing and specific Simple sequence repeats (SSRs) identification; **(C)** SSR polymorphism analyzing and primer designing. The black arrows represent the data flow.

Although there were a couple of pipelines, such as PolySSR (Tang et al., 2008) and SSRPoly (Duran et al., 2013), which may be used to identify PolySSRs in merely short sequences from EST datasets, none of them can handle the assembled large genome sequences mainly due to their adoption of a cluster-based strategy. Briefly, the pipeline of PolySSR (Tang et al., 2008) mainly consists of the three steps: (i) the EST sequences are clustered using CAP3 (Huang and Madan, 1999), and only the clusters with size between 2 and 500 are selected for subsequent analyses; (ii) then the C program named PolySSR together with Sputnik package are used for the prediction of SNPs and PolySSRs; and (iii) finally the Primer3 and CheckSSR implemented in PolySSR pipeline are used to design high-quality primers for PCR amplification. As for SSRPoly pipeline (Duran et al., 2013), it also adopts the similar cluster-based strategy like PolySSR, but differs in using a custom MySQL method and SSRPrimer for the PolySSR identification and primer designing. Both tools have succeeded in predicting PolySSRs in EST database, but they cannot further apply to large genome datasets from next generation sequencing (NGS), as both are not easy to complete their clustering steps in such long sequences or huge datasets. Moreover, the average sequence similarity of primer-binding regions among different species and/or individuals should be seriously taken into consideration to evaluate the levels of the confidence of SSR identification. However, both these two tools, PolySSR and SSRPoly, fail to provide solutions for this point. Thus we here developed an easy-to-use pipeline, CandiSSR (**Figure 1**), friendly enabling users to find putative PolySSRs not only from the transcriptome datasets but also from multiple assembled genome sequences of a given species or genus along with several comprehensive assessments. It would help researchers focus more on subsequent genetic studies on plants and animals of interest rather than aimlessly spending time on marker-screening experiments.

## Materials and Methods

### Data Accessibility

The genome sequences of six *Oryza* AA species are available at *Oryza* AA Genomes Database (http://www.plantkingdomgdb.com/). The genome sequences of 19 *Arabidopsis thaliana* accessions are available at: http://mus.well.ox.ac.uk/19genomes/. Rice and *Arabidopsis* PolySSR marker sources reported in this study are publicly available at: http://www.plantkingdomgdb.com/CandiSSR/index.html.

### Package Availability and Requirements

Project name: CandiSSR

Project home page: http://www.plantkingdomgdb.com/CandiSSR/

Operating system(s): Linux and UNIX

Programming language: Perl, and BASH

Other requirements: MISA (Thiel et al., 2003), BLAST (Altschul et al., 1997), Primer3 (Koressaar and Remm, 2007; Untergasser et al., 2012) and Clustalw (Thompson et al., 2002)

License: GNU General Public License v2

Any restrictions to use by non-academics: None

### Experimental Validation by PCR Amplifications

For each rice target SSRs, primers are automatically designed in our pipeline based on the Primer3 package (Koressaar and Remm, 2007; Untergasser et al., 2012). Additional information such as global similarities of the primer binding regions is also provided. Primers, which are completely conserved (100% global similarity of their primer binding region) in all six rice species, were selected, and the amplification specificity was further predicted by using the online tool Primer-BLAST (Ye et al., 2012) in the NCBI website (http://www.ncbi.nlm.nih.gov/). Genomic DNA for each rice sample was extracted by using the modified CTAB method (Doyle, 1987). Standard PCR amplifications were performed following the conditions below: 95°C for 1min; 30 cycles of 95°C for 30 s, 50–59°C for 20 s, and 72°C for 15 s; a final extension at 72°C for 1 min. PCR products were resolved by the electrophoresis on 8% non-denaturing polyacrylamide gels in 1x TBE (Tris-Borate-EDTA) buffer, and visualized by silver staining.

## Results and Discussion

### Implementation

The input files for CandiSSR are assembled sequences from a given species or genus in FASTA format. The major procedures to detect candidate PolySSRs in the pipeline are (**Figure 1**): (1) collect the assembled genome and/or transcriptome sequences of a given species or genus of interest; (2) rename their sequence header to avoid ambiguous description and subsequent error processing; (3) identify SSRs within the specified reference genome and/or transcriptome, and the mono-nucleotide repeat SSRs (MNRs) are removed; (4) retrieve the flanking sequences of the detected SSRs, and then align all the sequences except for those from reference genomes and/or transcriptomes to them using Blast (Altschul et al., 1997) without filtering low complexity sequences; (5) parse blast results and remove those low-quality hits that meet the criteria of <MI (Minimum Identity) and <MC (Minimum Coverage) using Bioperl package; (6) extract the non-reference sequence of each valid hit and then elongate a specified length from both sides; (7) search the specific reference SSRs within them; (8) remove those invalid searching items and yield the final list of SSRs; (9) analyze the SSR polymorphism and then filter out those low-quality PolySSRs matching standard deviation (SD) = 0 and Missing Rate (MR) > 50%; (10) output the final high-quality candidate PolySSRs; (11) calculate sequence similarities of flanking regions of the identified PolySSRs; and (12) design primer pairs and computationally assess the global similarity of primer binding regions for each PolySSR.

All these steps are automatically implemented in one Perl script, CandiSSR.pl, although the pipeline includes additional components implemented in Bash shell. When running the script, users can easily and rapidly obtain the detailed information of genome-wide and/or transcriptome-wide candidate PolySSRs of a given species or genus, including the SSR type, number of repeats, chromosome location, dispersion degree, MR, corresponding primer pairs, and their transferability. In addition, the flanking sequences with a specified length (-l option) of the finally identified PolySSRs are also generated so that users can simply use them to redesign the primer pairs for further PCR amplification or any other genetic studies depending on the demands of users.

### Candidate Polymorphic gSSRs in Rice and *Arabidopsis thaliana*

**FIGURE 2:**
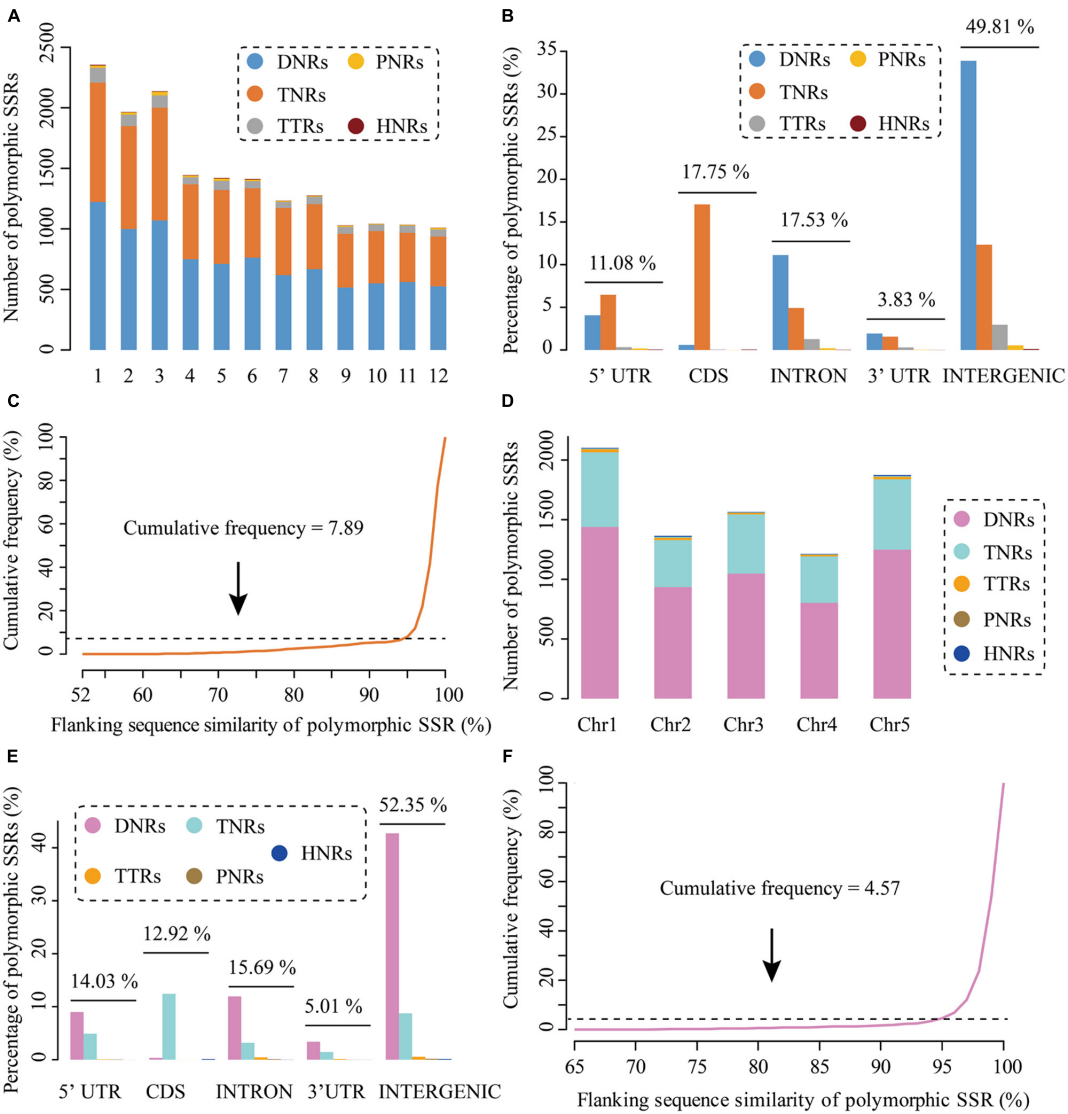
**Characteristics of the polymorphic SSRs (PolySSRs) identified in rice and *Athaliana thaliana*.** Chromosomal distribution **(A)**, genomic location **(B)**, similarity of flanking sequence **(C)** of rice PolySSRs; Chromosomal distribution **(D)**, genomic location **(E)**, similarity of flanking sequence **(F)** of identified PolySSRs of *A. thaliana*.

Rice is one of the most three important cereal crops together with maize and wheat for human consumption, providing staple food for more than half the world’s population (Khush, 1997, 2005; Goff et al., 2002). Up to now, although a number of PolySSRs have been developed in rice, more genetic markers are still required as the amount and their density in rice genomes are insufficient for satisfying the need of rice geneticists and breeders (Shen et al., 2004; Zhang et al., 2007). To prove the use of CandiSSR and enlarge the available PolySSRs in rice, in this study, we massively detected the rice candidate polymorphic gSSRs with the published genome sequences of six *Oryza* AA species that include *Oryza sativa* L. ssp. *japonica*, *O. nivara*, *O. glaberrima*, *O*. *barthii*, *O. glumaepatula*, and *O. meridionalis* (Goff et al., 2002; Zhang et al., 2014) using CandiSSR with default parameters. This identification took approximately 4.4 h on a Linux desktop computer that has 10 Gb memory and 2.13 GHz Dual-Core CPU. Consequently, a total of 17,374 rice PolySSRs with an average length of 17 bp were detected. These putative PolySSRs are predominately dispersed on the first three largest chromosomes (Chromosomes 1, 2, and 3; **Figure 2A**), showing a similar distribution with rice chromosome size. Di-nucleotide repeats (DNRs) are the most abundant repeat type (8,963; 51.59%) in rice PolySSRs, followed by tri-nucleotides (TNRs; 7,357; 42.34%), tetra-nucleotide (TTRs; 851; 4.90%), penta-nucleotides (PNRs; 163; 0.94%) and hexa-nucleotides (HNRs; 40; 0.23%). In addition, TNRs were mainly found in the coding regions (40.29%), while DNRs were principally distributed in intergenic (65.69%), intronic (21.56%), 5′-UTR (7.87%), and 3′-UTR (3.75%) regions (**Figure 2B**). Interestingly, all types of the identified rice PolySSRs had a similar distribution among different rice genomic regions except for TNRs, the percentages of which varied largely among different genomic regions (**Figure 2B**). Moreover, the average similarity of the flanking sequences of rice PolySSRs was 0.98, and approximately 92.11% of which was above 0.95, indicating a high potential of transferability of primer pairs that could be designed for these PolySSRs (**Figure 2C**). Meanwhile, a total of 16,556 PolySSRs, accounting for ∼95% of all the rice PolySSRs identified, can be designed with primers. In comparison, the determination of the candidate polymorphic gSSRs in *A. thaliana* with a total of 19 *Arabidopsis* genomes (Gan et al., 2011) spent about 9.7 h with the same Linux device due to a large total sequence size (∼2.23 Gb) from more screened genomes. As a result, a total of 8,119 putative PolySSRs were detected in *A. thaliana*. The average length of *A. thaliana* PolySSRs was 18 bp, which is slightly larger than that of rice. Like rice, the chromosomal distribution of *A. thaliana* PolySSRs is also consistent to chromosome size, and most of them are intensely distributed on chromosomes 1, 5, and 3 (**Figure 2D**). Intergenic region was the dominant genomic region to cover nearly 52.35% of these PolySSRs. Similarly, the majority of the TNRs were located within the protein-coding regions, whereas DNRs were massively distributed within the intergenic, intronic, 5′-UTR, and 3′-UTR regions (**Figure 2E**). The average similarity of the flanking sequences of the *A. thaliana* PolySSRs was 0.99 and 95.43% of them were >0.95, which is considerably greater than that of rice (**Figure 2F**). Overall, the PolySSRs reported here have significantly expanded the number of molecular markers publicly available for rice and *A. thaliana* in the databases. More importantly, researchers can easily use this pipeline to rapidly generate numerous high-quality usable PolySSRs for a target genus (e.g., *Oryza*) or species (e.g., *A. thaliana*), which will greatly accelerate relevant genetic studies.

### Rapid Identification of Tea Polymorphic EST-SSRs

**FIGURE 3:**
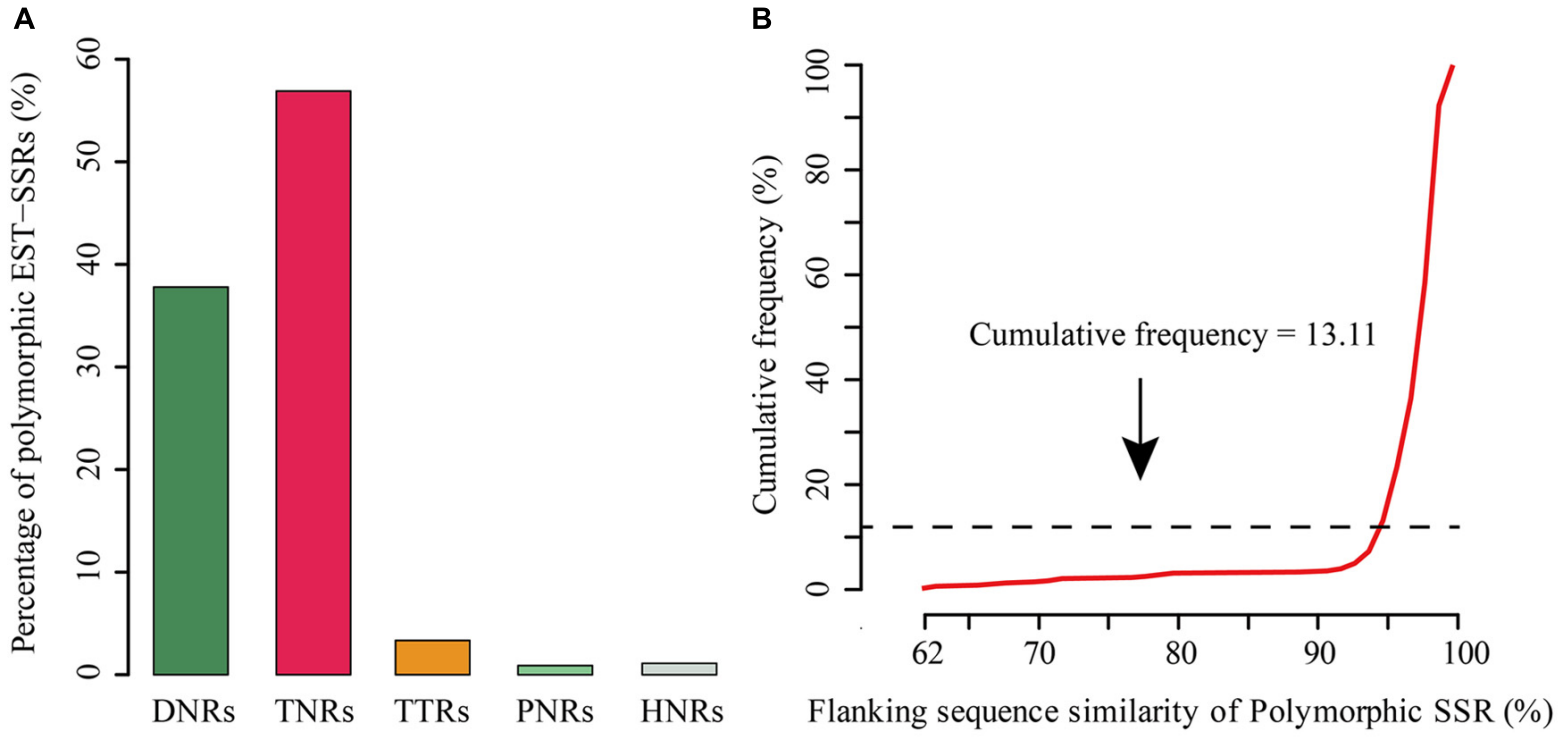
**Overview of the candidate polymorphic EST-SSRs detected in the *Camellia* species.** **(A)** Distribution of the polymorphic EST-SSRs; **(B)** Distribution of the flanking sequence similarity of PolySSRs.

Expressed sequence tag-SSR is another type of SSR that specifically derived from transcribed gene regions of a given organism, and therefore, they may be associated with some important traits or pathways (Kaur et al., 2011). In this study, as another case study, we identified the putative tea polymorphic EST-SSRs with four published transcriptomes in the genus *Camellia*, including *Camellia sinensis*, *C. taliensis*, *C. oleifera*, and *C. reticulata* (Shi et al., 2011; Xia et al., 2014; Zhang et al., 2015). With the same Linux system above-described to detect rice and *A. thaliana* polySSRs, the identification of tea polymorphic EST-SSRs using CandiSSR with default parameters took no more than 5.85 min. Finally, a total of 450 polymorphic EST-SSRs were generated with an average length of 17 bp. Of them, TNRs were the most abundant type (256; 56.89%), followed by DNRs (170; 37.78%), TTRs (15; 3.33%), HNRs (5; 1.11%), and PNRs (4; 0.89%) (**Figure 3A**). Among DNRs, GA/TC (31.18%) was quite dominant, followed by AG/CT (26.47%) and TA/TA (17.65%). ACC/GGT (11.33%) was the most abundant motif for TNRs. The flanking sequence similarity of over 86.89% tea polymorphic EST-SSRs was greater than 95% (**Figure 3B**). In addition, primer pairs could be successfully designed for a total of 440 (97.78%) PolySSRs. To the best of our knowledge, although there are much more SSRs that were previously reported in the genus *Camellia*, relatively fewer polymorphic loci have been identified (Ma et al., 2010; Wen et al., 2012; Tong et al., 2013). Thus, the PolySSRs reported here will be particularly valuable for the germplasm characterization and utilization in the genus *Camellia*.

### Experimental Validation of 10 Randomly Selected Rice Polymorphic SSRs

**FIGURE 4:**
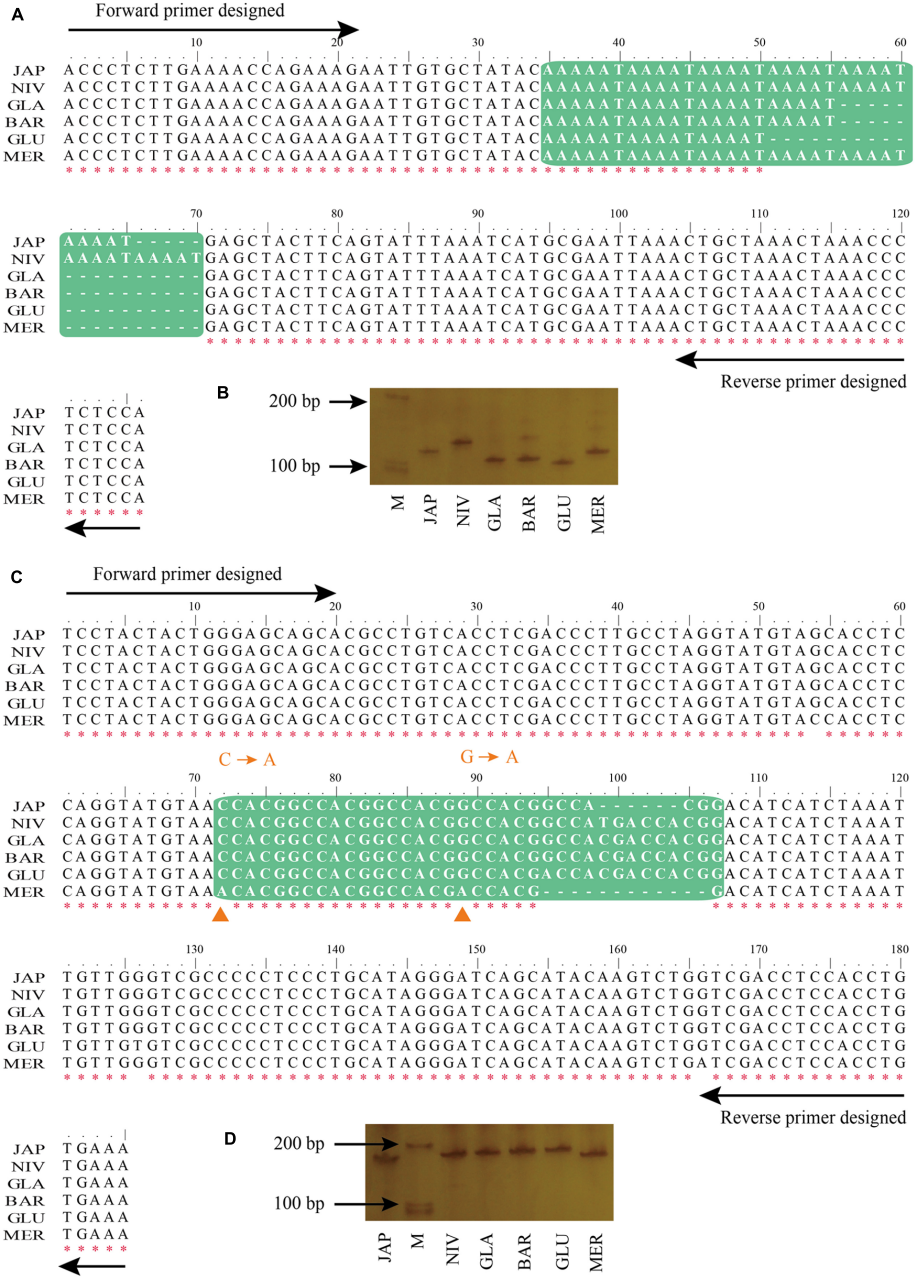
**Detailed information of the two randomly selected rice PolySSRs.** **(A)** Multiple sequence alignment (MSA) for flanking regions of CPSSR_9 detected by CandiSSR; **(B)** PCR validation of polymorphic status for CPSSR_9; **(C)** MSA for flanking regions of CPSSR_4933; **(D)** PCR validation of polymorphic status for CPSSR_4933. Green box represents the PolySSRs among different rice species, while red asterisk indicates the clustal consensus for each position. Orange triangle denotes the base variations among rice species.

To experimentally validate the PolySSRs detected by using CandiSSR, we randomly selected 10 rice PolySSRs that cover all MS types, two for each type (TNR, DNR, TTR, HNR, and PNR), for the PCR experiments (**Table 1**). The detailed PCR results were presented in **Supplementary Figure S1**. All of the tested primer pairs were successfully amplified, showing a good transferability of these primer pairs among these six rice species. Additionally, nine of the 10 tested PolySSRs were unquestionably confirmed to be polymorphic among these six rice species except for SSR CPSSR_10489, which was amplified with multiple DNA bands, indicating a high accuracy rate of 90% by using this pipeline. In most of the cases, the lengths of PCR products are solely affected by the number of SSR repeats that can be easily determined by electrophoresis experiments. For instance, CPSSR_9 was a typical case that the length of PCR products is concordant with the number variation of SSR repeats (**Figures 4A,B**). Note that both Indels and base substitutions may occasionally exist in the flanking regions of the detected SSRs that may complicate the results of experimental validation. For example, CPSSR_4933 had five repeats for the motif “CCACGG” in the *japonica* rice but showed a shorter PCR product than that of *O. nivara* (four repeats), *O. glaberrima* (four repeats), *O. barthii* (four repeats) and *O. glumaepatula* (three repeats), mainly because the flanking regions of the *japonica* rice contained a 6 bp deletion within the fifth repeat motif (**Figures 4C,D**). Besides, CPSSR_4933 had two substitutions in the “CCACGG” target regions of *O. meridionalis*, resulting in only one continuous repeat of “CCACGG” retained in this species.

**Table 1:** Primer pairs of the candidate rice polymorphic SSRs (PolySSRs) employed for PCR validation.

CandiSSR ID	Forward (5′->3′)	Reverse (5′->3′)
CPSSR_9	ACCCTCTTGAAAACCAGAAAGA	TGGAGAGGGTTTAGTTTAGCAGT
CPSSR_361	TTCAGGTACTATGCGAGCGT	CTGCTCTGATCGCTGTTCCA
CPSSR_2506	GTCCAGGTGTCTGCTTCCAT	GCCCTCTCGTGAGCTCTAAG
CPSSR_4482	ACCACAGCACGGAGAATCAG	GGAGCGGAAAGGGTTGGATT
CPSSR_4933	TCCTACTACTGGGAGCAGCA	TTTCACAGGTGGAGGTCGAC
CPSSR_9097	TTTCCAGTTGTTCGCTTCGC	TTTCCGTCGTCGATCCACTC
CPSSR_10489	AGTTTGTGTCGGGGAGCAAA	CATCTCTCTCCGCGATCGTC
CPSSR_10941	TGAGGTGTTCTTGGACGACA	TGCTGCTGTTCTTGTGTTGC
CPSSR_13442	AGCCATTGTTATGCAAACGGT	TGTTTTCCCACGATGAGACG
CPSSR_14617	AGAGGCCGTGAGAATTTCCG	GCACTGTACCATAGTTTTGGACA


*Specific primer pairs of the 10 randomly selected PolySSRs are shown, which were automatically designed by using CandiSSR based on the Primer3 software.*

### Dependence of CandiSSR Running Time on the Number of Organism Samples

**FIGURE 5:**
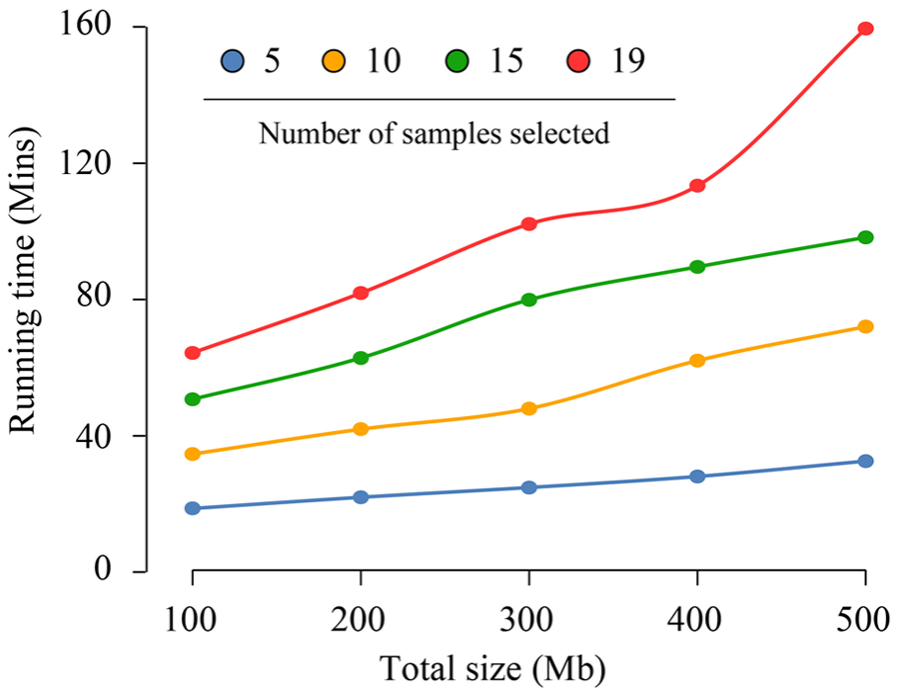
**Running time changes of CandiSSR depending on the number of samples.** The blue, orange, green, and red lines show the running time changes for 5, 10, 15, 19 *Arabidopsis* genomes, respectively. As shown in the figure, the running time of the pipeline is largely affected by the number of samples and partially related to the total size of the assembled sequences.

The running time of this pipeline was 9.7 h to identify putative PolySSRs in *A. thaliana* with approximately 2.23 GB assembled data from the 19 *A. thaliana* genomes. Such a result indicates that the pipeline can handle as many as 19 *A. thaliana* genomes harboring nearly 230 Mb data per hour. However, it is not clear whether the total size of the assembled sequences or the number of genome samples essentially determine the whole running time of CandiSSR. To make this clear, we chose the Col-0 genome of *A. thaliana* (∼119 Mb) as reference and estimated the running times of CandiSSR with different data sets that produced from the 18 *A. thaliana* genomes. As shown in **Figure 5**, each line demonstrated the change of running time depending on the differences of total sizes of assembled sequences for a specific sample number. Obviously, the running times for different total sizes of assembled sequences change slightly when fewer samples are provided, otherwise they vary considerably. For example, the running time ranged from 18.7 to 32.6 min for five samples with total lengths from 100 to 500 Mb (**Figure 5**). On the contrary, running with a total of 19 samples containing the same size of datasets took increased times between 64.3 and 159.5 min. In addition, the average running times for datasets having differently total sizes of assembled sequences for 5, 10, 15, and 19 samples were 25.2, 51.7, 76.2, and 104.2 min, respectively, suggesting that nearly 26.3 min should be taken into consideration for processing each increased five samples. On the other hand, the average running times for datasets containing 100, 200, 300, 400, and 500 Mb were 42.1, 52.1, 63.7, 73.2, and 90.6 min, respectively. This result suggests that processing additional 100 Mb data merely needs approximately extra 12.1 min, which is considerably shorter than the time for handling every five added samples. Hence, we may conclude that the running time of CandiSSR is largely affected by the total number of samples but is partially related to the total size of the assembled sequences.

## Conclusion

Using CandiSSR, users can efficiently identify numerous PolySSRs from multiple assembled sequences of a target genus or species. These genome and/or transcriptome sequences can be assembled from a number of sequencing strategies. Therefore, this pipeline can help the research community to easily collect plentiful PolySSRs that will undoubtedly accelerate genetic studies on and enhance breeding programs of plants and animals of great interest.

## Author Contributions

L-ZG and E-HX conceived and designed the experiments. E-HX developed the pipeline and drafted the manuscript. Q-YY wrote the module for primer designing. J-JJ and L-PZ performed the seed germination and extracted the DNA samples. Q-YY and H-BZ performed the experimental validation. L-PZ, J-JJ, and H-BZ revised the manuscript. All authors read and approved the final manuscript.

## Conflict of Interest Statement

The authors declare that the research was conducted in the absence of any commercial or financial relationships that could be construed as a potential conflict of interest.

## Supplementary Material

**FIGURE S1 | Experimental validation of the 10 randomly selected PolySSRs in rice.** 10 PolySSRs covering all MS types, two for each type, are randomly selected for PCR validation. The detailed information of primers pairs are given in **Table 1**. PCR products were resolved by the electrophoresis on 8% non-denaturing polyacrylamide gels and visualized by silver staining. Lanes for each SSR are labeled, and their templates followed by the order of “*Oryza sativa* L. ssp. *japonica*, *O. nivara*, *O. glaberrima*, *O. barthii*, *O. glumaepatula*, and *O. meridionalis.*”
