# Supplementary figures and images for: CandiSSR: An Efficient Pipeline used for Identifying Candidate Polymorphic SSRs Based on Multiple Assembled Sequences
Source: Front Plant Sci. 2016 Jan 7;6:1171. doi: 10.3389/fpls.2015.01171 (PMC4703815; doi:10.3389/fpls.2015.01171)

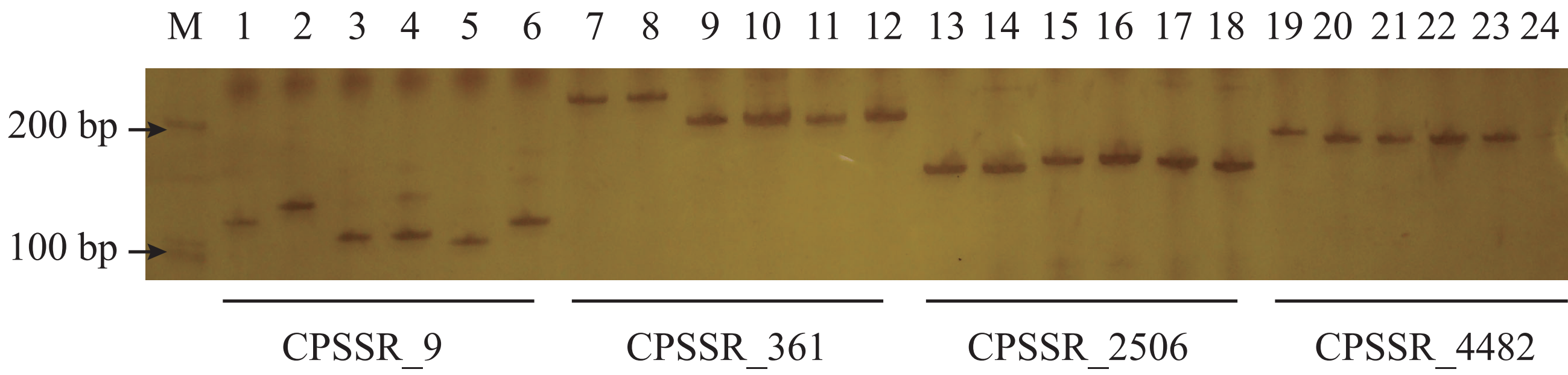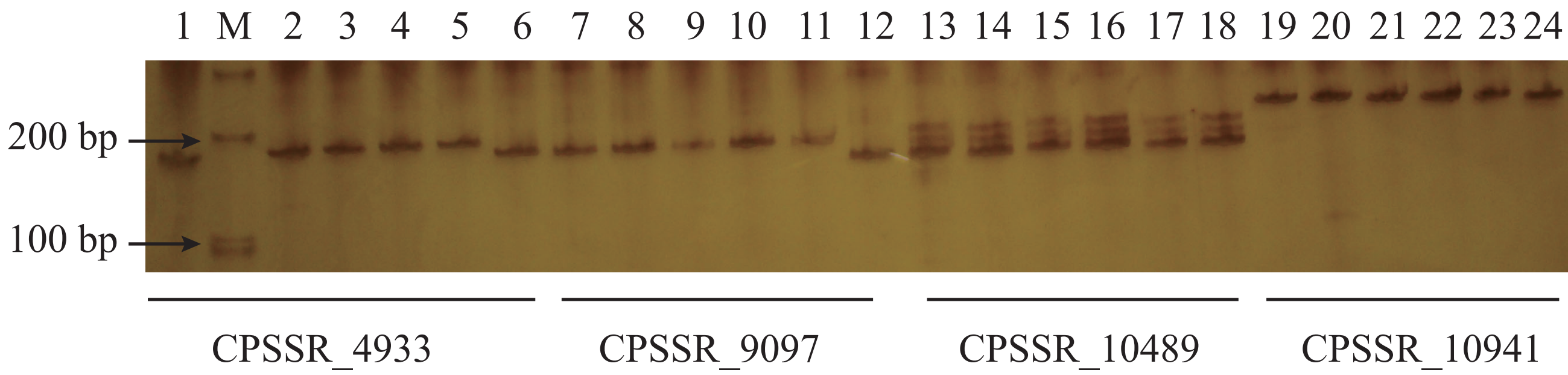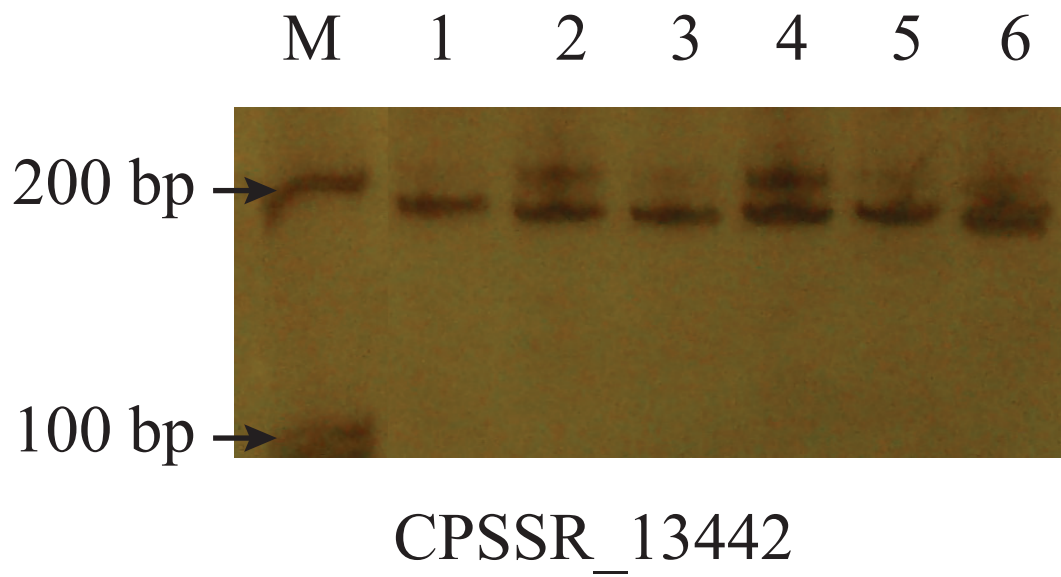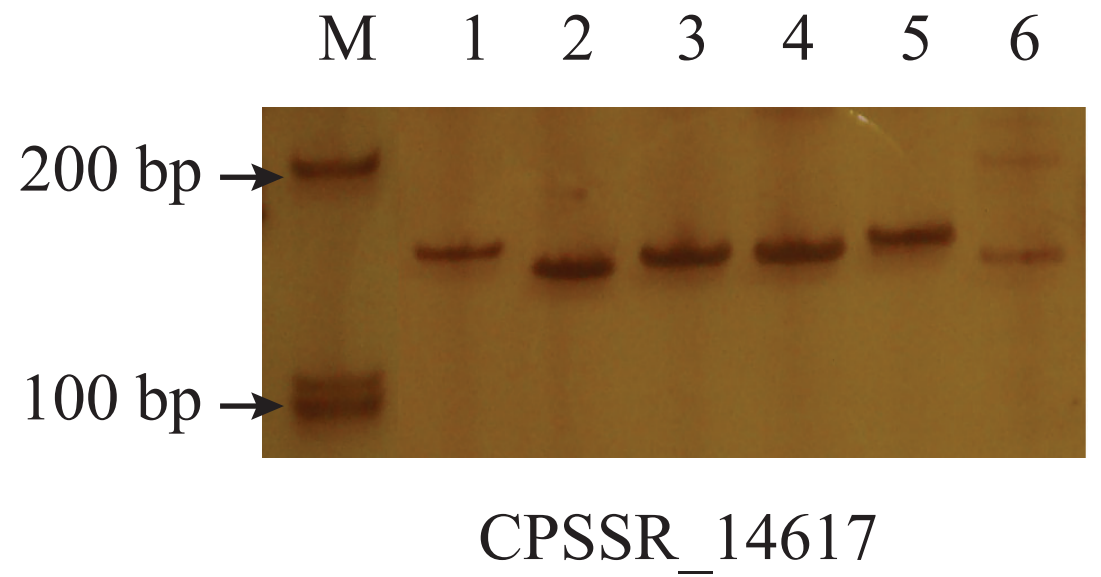

Supplement: Supplementary file 1 [file Image_1.PDF]
